# Memorial of Dr. John Bell, and Rejoinder of Prof. S. Jackson

**Published:** 1850-11

**Authors:** 


*Memorial of Dr. John Bell, and Rejoinder of Prof. S. Jackson.*—Some weeks since we were favored by a Memorial addressed to the Trustees of the University of Pennsylvania, by Dr. John Bell. It appeared from the Memorial that Dr. B. had been an unsuccessful candidate for the vacancy in the Medical Department of the University occasioned by the resignation of Prof. Chapman. It was evidently written under a poignant sense of injustice for which the author held certain members of the Society responsible, attributing to them the exertion of undue influence, in opposition to his appointment, with the Board of Trustees. The publication of the Memorial must have been deemed unfortunate by the friends of Dr. B., more especially as he did not confine himself to a rehearsal of his supposed wrongs, but endeavored to retaliate by personal attacks with that most dangerous of all weapons, *satire*—a two-edged sword which is apt to wound those who wield it. Whether Dr. B. has just occasion for feeling aggrieved, we do not presume to judge. In the choice of a Professor, the Managers of the University of Pennsylvania would be expected to select from the list of candidates, the one whose labors as a teacher, would, in their estimation, be most likely to prove successful. The interests of the School and of the Faculty, of course, dictate this policy, and not less, also, the interests of Medical Education. If, in pursuing this line of conduct, the result conflicted with the supposed personal claims of any individual, whatever chagrin that individual may have felt, it would certainly have been wiser and in better taste, to have brooded over it in silence, than to seek to enlist the public in his private feelings of disappointment and resentment. We are constrained to say that our sentiments of great respect for Dr. Bell, led us to regret deeply that some judicious confidential adviser had not stepped between him and the printer, and placed his veto on the publication of a document which, in the sober moments of the author, will be deprecated by himself as sincerely as by those who esteem him most.

Within a few days we have received a rejoinder to the Memorial of Dr. B., with the initials of the name of Prof. Samuel Jackson, appended. The latter retorts in the mode of warfare which had been unwarily provoked, and few writers have the art of directing satire and ridicule with greater effect. His pen is a paixhan of the first magnitude. But Dr. Johnson would perhaps have said of this effort, as of one of the productions of Goldsmith, "It is a foolish thing, exceedingly well done." Personal quarrels are scarcely less defensible when carried on by the artifices of rhetoric, than when the combatants resort to the bowie knife and pistol, and the latter has the advantage of settling disputes more speedily.

Respecting, as we do, in common with the medical profession, both the distinguished Professors who have been betrayed by excited feelings into an attitude toward each other of intellectual pugilism, we concur most heartily in the wish expressed by our contemporary, the New-York Medical Gazette, that the "old warriors will bury the hatchet and smoke the calumet of peace."

